# Pneumonia in Nervous System Injuries: An Analytic Review of Literature and Recommendations

**DOI:** 10.7759/cureus.25616

**Published:** 2022-06-02

**Authors:** Zohreh Erfani, Hesan Jelodari Mamaghani, Jeremy Aaron Rawling, Alireza Eajazi, Douglas Deever, Seyyedmohammadsadeq Mirmoeeni, Amirhossein Azari Jafari, Ali Seifi

**Affiliations:** 1 Faculty of Medicine, Tehran University of Medical Sciences, Tehran, IRN; 2 Department of Medicine, University of Texas Health Science Center at San Antonio, San Antonio, USA; 3 Department of Radiology, University of Texas Health Science Center at San Antonio, San Antonio, USA; 4 Department of Pulmonary and Critical Care Medicine, Brooke Army Medical Center, San Antonio, USA; 5 Student Research Committee, School of Medicine, Shahroud University of Medical Sciences, Shahroud, IRN; 6 Department of Neurosurgery, University of Texas Health Science Center at San Antonio, San Antonio, USA

**Keywords:** neuro-icu, demyelinating diseases, neuromuscular diseases, intracerebral hemorrhage, traumatic brain injury, subarachnoid hemorrhage, stroke, neurointensive care unit, pneumonia

## Abstract

Pneumonia is one of the most common complications in intensive care units and is the most common nosocomial infection in this setting. Patients with neurocritical conditions who are admitted to ICUs are no exception, and in fact, are more prone to infections such as pneumonia because of factors such as swallow dysfunction, need for mechanical ventilation, longer length of stay in hospitals, etc. Common central nervous system pathologies such as ischemic stroke, traumatic brain injury, subarachnoid hemorrhage, intracerebral hemorrhage, neuromuscular disorders, status epilepticus, and demyelinating diseases can cause long in-hospital admissions and increase the risk of pneumonia each with a mechanism of its own. Brain injury-induced immunosuppression syndrome is usually considered the common mechanism through which patients with critical central nervous system conditions become susceptible to different kinds of infection including pneumonia. Evaluating the patients and assessment of the risk factors can lead our attention toward better infection control in this population and therefore decrease the risk of infections in central nervous system injuries.

## Introduction and background

Nosocomial infections are one of the most common adverse consequences in patients with major neurological damage admitted to the intensive care units [1]. About 36% of patients admitted to neurointensive care units (neuro-ICUs) for a period exceeding 48 hours develop nosocomial infections with pneumonia being the most common type of infection [2]. Urinary tract infections, bacteremia, and intracranial infections such as ventriculitis and meningitis account for the remaining bulk of nosocomial infections in neuro-ICUs [3]. These infections can lead to a higher mortality rate in critically ill patients in addition to generating higher expenses imposed on healthcare systems as well as worsening patient outcomes [4-8].

Ventilator-associated pneumonia (VAP) is the most common type of pneumonia in patients admitted to neuro-ICUs. By definition, it is pneumonia that develops in mechanically ventilated patients at least 48 hours post endotracheal intubation without evidence of preexisting infection; it is caused by aspiration of oropharyngeal secretions around the endotracheal cuff and into the tracheobronchial tree [9]. Nosocomial pneumonia in ICU patients leads to an increased length of hospital stay and an increase in mortality. Alarmingly, in the case of brain-injured patients, sepsis and VAP are the major causes of death during hospitalization in a neuro-ICU with pneumonia. Additionally, VAP can cause acute respiratory distress syndrome (ARDS) in neurocritical patients with traumatic brain injury (TBI) [10].

Patients with subarachnoid hemorrhage (SAH), stroke, and TBI all require intensive care and may warrant admission to neuro-ICUs where they may become susceptible to nosocomial infections such as pneumonia. The highest association with nosocomial infections has been observed in patients with a subdural hematoma and intracerebral/intraventricular hemorrhage (IVH) with incidence rates as high as 21.3 and 21.1 per 1000 neuro-ICU days, respectively [11]. It has been shown that the proportion of mortality due to medical complications including nosocomial infections in SAH patients is comparable to the proportion of mortality due to the direct effects of the initial hemorrhage, rebleeding, and vasospasm in these patients collectively [12]. In addition, the prolonged length of stay (LOS) that occurs commonly in these patients can lead to more nosocomial infections which in turn may further increase the LOS and create a vicious cycle that can affect mortality, morbidity, and associated costs imposed on the patients and the system [7,13-15].

In patients with stroke, a significant infection rate of up to 33% has been observed with pneumonia comprising 10-25% of those infections seen in neuro-ICUs. This rate is comparable to that seen in general ICUs [16-18]. However, some newer studies suggest even higher rates of general infections, up to 45% of stroke patients with 28% of those accounted for by pneumonia. This, in turn, is significantly associated with mortality in post-stroke patients in neuro-ICUs [19].

Despite all these statistics, the exact pathophysiology and pathogens involved in pneumonia in central nervous system (CNS) injuries are still being studied. Reaching a conclusive approach to the prevention and treatment of pneumonia in this background can decrease mortality, improve outcomes, and diminish the costs imposed on patients and the healthcare system. In this analytical review, we aim to go over updates on pneumonia in neurological injuries, predisposing pathophysiology, differences with general ICUs, and treatments.

## Review

Pathophysiology of pneumonia in nervous system injuries

Pneumonia in the ICU setting is the most common cause of morbidity in patients leading to worsening of the medical condition, prolonged ICU stays, increased need for invasive interventions, and increased risk of hospital readmission [20]. Risk factors for hospital-acquired pneumonia (HAP) include Glasgow Coma Scale (GCS) less than 8, mechanical ventilation, impaired airway reflexes, supine positioning, aspiration, preexisting diseases like chronic obstructive pulmonary disease (COPD), burns, prolonged ICU stay, use of positive end expiratory pressure (PEEP) during mechanical ventilation, high disease severity, multiple organ dysfunction, older age, prior administration of antibiotics, malnutrition, use of the nasogastric tube, use of paralytic agents, male gender, enteral feeding, immunosuppression, and trauma [21,22]. The common pathogens that are involved are *Staphylococcus aureus*, *Klebsiella pneumoniae*, *Pseudomonas aeruginosa*, *Streptococcus pneumoniae*, and *Enterobacter aerogenes* [23]. However, the nature of the critical conditions in CNS leads to higher susceptibility to developing pneumonia compared to general ICUs, due to factors such as brain injury-induced immune dysregulation and immunosuppression, high prevalence of dysphagia, and placement of external ventricular drains (EVDs) [24].

Brain injury-induced immune dysregulation is primarily caused by an elevated inflammatory response which leads to central and peripheral production of chemokines, proinflammatory cytokines, and cell adhesion molecules in these patients [1,25-26]. Production of these cytokines and the development of the inflammatory response is a crucial part of clearing cellular debris in the CNS following an injury; however, chronic and prolonged inflammation response can lead to dysregulation in the immune system [27-29]. This dysregulation following traumatic events, brain surgery, SAH, or spinal cord injury (SCI) is called brain injury-induced immunodepression syndrome, and when it is after stroke, it is named stroke-induced immunodepression syndrome (SIDS)) [30-32]. SIDS is considered to be biphasic. The first phase starts as soon as 12 hours after the initial injury with early transient activation lasting up to 24 hours, and the second phase consists of a systemic immunodepression that can last for several weeks [32-34].

Immunosuppression also occurs due to prolonged catecholamine release. Following a brain injury, the hypothalamic-pituitary axis is activated along with the sympathetic nervous system and, as a result, catecholamines are released. This can also trigger the inflammatory response as previously discussed [35-37]. In addition, β2-adrenergic receptors help mediate the response [38]. The increase in sympathetic system activity and high catecholamine levels and the consequent immunosuppression are highly associated with infections in brain injury patients [39-41]. Norepinephrine, being the main neurotransmitter in the sympathetic nervous system, is released into the lymphoid tissue and modulates the function of immune cells [42]. This modulation includes increased lymphocyte apoptosis, lymphocyte depletion, impaired monocyte function with diminished human leukocyte antigen, antigen D related (HLA-DR) expression, decreased activity of natural killer (NK) cells, shift from T-helper 1 (Th1) to Th2 cytokine production, increased release of cytokines from activated T-cells, increased production of tumor necrosis factor-alpha (TNF-α) and interferon-gamma (IFN-γ) by spleen and blood, increased release of platelets from the spleen, and a higher ratio of CD4/CD8 T-cells [35-37,41,43-44]. The subsequent immunodepression is largely attributed to an increase in systemic regulatory T-cells, decreased IFN-γ production, impaired response by NK cells and T-cells, with insufficient activation of phagocytes at the infection site [37,45].

**Figure 1:**
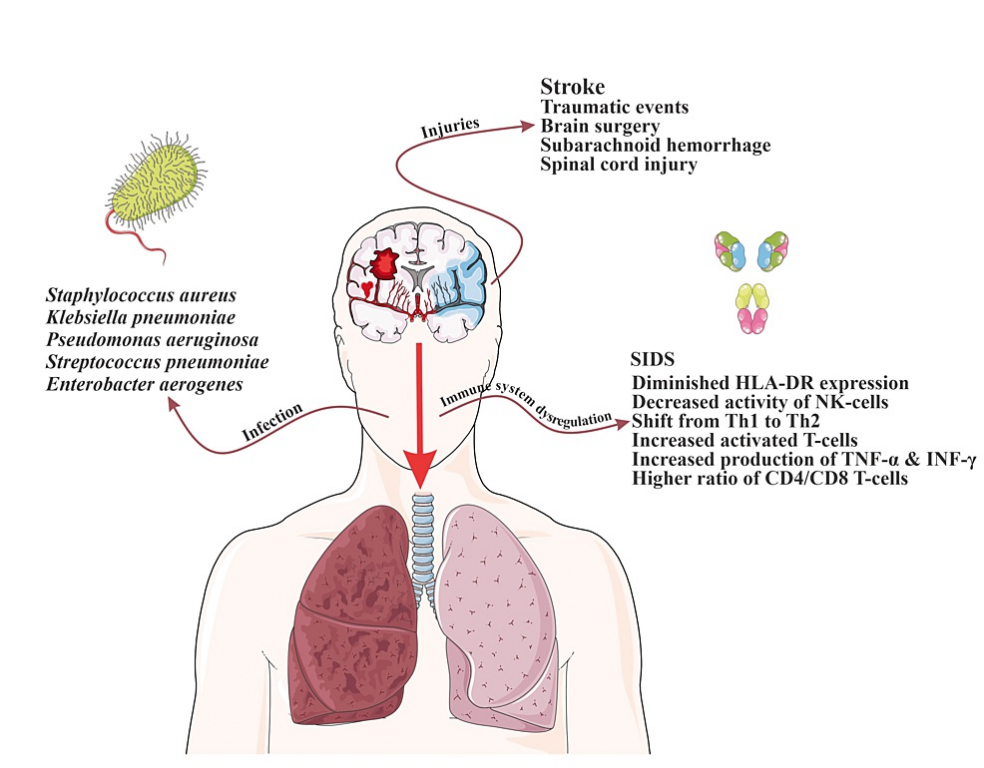
The Main Pathophysiology of Pneumonia in CNS Injuries SIDS: stroke-induced immunodepression syndrome; HLA-DR: human leukocyte antigen, antigen D related; TNF-α: tumor necrosis factor-alpha; IFN-γ: interferon-gamma; Th: T-helper This figure was drawn using images from Servier Medical Art (https://smart.servier.com) licensed by a Creative Commons Attribution 3.0 Unported License.

Some studies have found that injuries within the right frontal region and putamen increase susceptibility to infections [46]. Insular cortex strokes correlate with the highest risk of pneumonia due to their association with excessive sympathetic stimulation [43]. The volume of the infarct is also a major factor in determining the risk of infection due to varying amounts of resultant immunomodulation [47]. Other factors aside, the autonomic shift is considered to be the main element in infection development regardless of the severity of the injury [48]. Baroreceptor sensitivity is also found to be decreased in patients with acute stroke, indicative of a shift toward sympathetic system activation [48]. This decreased sensitivity is significantly associated with worse outcomes and can predict infectious complications in intracranial hemorrhage, with IVH in particular (Figure 1) [48-50].

Pneumonia in specific CNS injuries

Ischemic Stroke

In regards to ischemic strokes, pneumonia is the most common post-event infection [19]. Stroke-associated pneumonia (SAP) has a significant effect on prognosis, LOS, and healthcare costs [1,51].

In 2015, the Stroke Consensus Group defined SAP as a lower respiratory tract infection occurring within the first 7 days post-stroke in non-ventilated patients with respiratory tract infections occurring after that point defined as HAP. Admittedly, there was no effort made to differentiate SAP from community-acquired pneumonia (CAP). One prospective observational study of patients in a university-based neuro-ICU found that patients diagnosed with SAP (using diagnostic criteria provided by CDC guidelines in 1988) spend, on average, six more days in the neuro-ICU compared to their non-diseased counterparts. In their study, they did identify *S. aureus*, Klebsiella, Enterobacter species, and *Escherichia coli* as the causative organisms [16]. The following criteria in Tables 1-2 are currently recommended by the Stroke Consensus Group for the diagnosis of SAP [52].

**Table 1:** Stroke-Associated Pneumonia Criteria [52]

Requirement	Findings
At least one of the following	Altered mental status without any other recognized reason in age≥ 70
	Leukopenia (<4000 WBC/mm^3^) or leukocytosis(>12000 WBC/mm^3^)
	Fever(>38°C) without any other reason
At least two of the following	New onset or worsening cough, dyspnea, or tachypnea (respiratory rate>25/min)
	New onset of purulent, change in character of sputum over a 24-h period, increase respiratory secretions, or increase suctioning requirements
	Rales, crackles, or bronchial breath sounds
	Worsening gas exchange (e.g., O₂/FIO₂≤240), increased oxygen requirements)
And ≥2 serial chest radiographs with at least one of the following	New or progressive and persistent infiltrate, consolidation, or cavitation
	In patients without underlying pulmonary or cardiac disease, one definitive chest radiograph is acceptable

**Table 2:** Stroke-Associated Pneumonia Diagnosis [52]

Type of Diagnosis	Criteria
Probable stroke-associated pneumonia	All criteria met, but initial chest X-ray and serial/repeat nonconfirmatory (or not undertaken), and no alternative diagnosis or explanation
Definitive stroke-associated pneumonia	All criteria met, including diagnostic chest X-ray changes (on at least one)

Studies show that procalcitonin (PCT) may be a useful tool in aiding the diagnosis of pneumonia in a newborn intensive care unit (NICU) as well since it elevates quickly compared to WBC and C-reactive protein (CRP), is elevated in bacterial (but generally not viral or fungal) infections, and downtrends rapidly after treatment in patients with normal renal function. However, it is still controversial if PCT can be applied as a diagnostic measure, and not merely as a tool for de-escalation of antibiotics [53].

Known risk factors for SAP include dysphagia, higher National Institutes of Health Stroke Scale (NIHSS), non-lacunar basal-ganglia infarction, age, large middle cerebral artery (MCA) infarction, multiple hemispheric or vertebrobasilar infarction, mechanical ventilation on admission, and impaired vigilance. The presence of intubation also increases the risk of pneumonia independently of the presence of known aspiration [54]. This may be due to the loss of time from stroke onset to treatment because of intubation [54]. An additional factor may be the variation in blood pressure during the induction of anesthesia for intubation, which can result in hypoperfusion and subsequent further damage to the ischemic penumbra [54]. Conversely, a lower risk of SAP was seen in small-vessel occlusions [55]. In addition, the high association between the insular cortex and the sympathetic nervous system causes higher sympathetic system activation in case of insular cortex infarcts and as a result, stronger brain injury-induced immunosuppression occurs which increases the chance of pneumonia in these patients [43]. Immunomodulation severity also varies in proportion to the volume of infarcts [47]. More massive infarcts give rise to stronger immunomodulation and as a result a higher chance of pneumonia development [47]. In regards to predicting the development of SAP, a high neutrophil to lymphocyte ratio (NLR) has shown promise as a predicting agent and could help avoid this unfavorable consequence in stroke patients [56].

Aside from the immune dysregulation and immunosuppression resulting from the brain injury, there are other reasons for explaining the pathophysiology of SAP. Studies show that impaired levels of consciousness and dysphagia play important roles in SAP development [57]. In particular, swallowing mechanism dysregulation is not uncommon in stroke patients and can lead to aspiration. Several studies have noted a correlation between this abnormality and abnormal dopamine transmission. Specifically, in ischemic stroke patients there was noted to be a decreased level of substance P in those patients who developed aspiration pneumonia; previous studies in guinea pig models were able to demonstrate inhibition of swallow function with blocking of D1 dopamine receptors and resultant decreased level of substance P [58-60]. Another study attempting to elucidate the etiology of SAP predisposition in stroke patients utilized monocytic HLA-DR expression as a marker for SIDS and found that risk was increased when HLA-DR levels were below the median. The same study noted that higher levels of lipopolysaccharide-binding protein (LBP), an acute-phase reactant, correlated with increased risk of SAP; however, these two surrogates were found to have better utility in the prediction of SAP development if used earlier in the course of the disease [61].

Traumatic Brain Injury

Patients with TBI are found to develop a nosocomial infection at a rate of 41%, with pneumonia being most common with an incidence rate of 30% or more [1,62-65]. Several risk factors have been observed to play a major role in infection development in patients with TBI including surgical intervention, prolonged hospitalization, damage to the CNS, CSF leak, nasal carriage of *S. aureus*, use of barbiturates, and the need for intubation and mechanical ventilation [65,66]. In addition, patients with these characteristics were more likely to develop pneumonia: male gender, younger age, intubation on the scene or in the emergency department, lower GCS score, longer mechanical ventilation, higher injury severity score, and further injuries to the brain [67]. VAP has been observed to have bimodal development with typical onset between days 5 and 11 of ICU stay [68,69]. Early onset VAP has been associated with the presence of thoracic injury, high injury severity score, and coma on admission. Similarly, independent predictors of late-onset VAP include age, high injury severity score, and coma duration [69]. Furthermore, the aforementioned brain injury-induced immune deficiency is also a major factor in the development of infections in patients with TBI. A significant inflammatory response is independently created in TBI situations, and prolongation of this inflammatory response may lead to pneumonia and other infections [28,35,70-71].

Common pathogens involved in VAP diagnosed in TBI patients are methicillin-sensitive *S. aureus* (MSSA), *Haemophilus influenzae*, *S. pneumoniae*, and Acinetobacter species [69,72]. In regard to the relative burdens of disease caused by the specific pathogens, one particular study found that the typical organisms encountered varied depending on whether the VAP was classified as occurring early or late. The early versus late classification used in this particular study was based on American Thoracic Society guidelines and was defined as having been diagnosed within the first four days of intubation versus on or after the fifth day of intubation. Within the confines of their cohort study, the organisms responsible for the early onset of VAP were MSSA (46%), Enterobacter species (20%), *S. pneumoniae* (9%), *P. aeruginosa* (9%), *H. influenzae* (7%), and methicillin-resistant *S. aureus* (MRSA) (2%). The late-onset VAP group was noted to have higher rates of both MRSA (11%) and *P. aeruginosa* (16%) [73]. A single-institution prospective, observational cohort study focusing specifically on VAP noted that, of their culture-positive VAP cases, 62% were monomicrobial and 38% were polymicrobial. Although they did not differentiate which organisms were found in monomicrobial versus polymicrobial cultures, they did quantify the percentages of each organism found in total. 31% of isolates were accounted for by *S. aureus*, 28% by *H. influenzae*, 15% by non-classified gram-negative bacteria, and 8% by non-classified gram-positive species [74].

Several morbidities are observed in patients with TBI who develop nosocomial infections which can lead to worse outcomes, including longer ICU and hospital LOS, higher organ system dysfunction, longer mechanical ventilation, and worse neurologic outcome seen at follow-up [74-77]. Although the influence of nosocomial infections in patients with TBI on mortality is yet to be elucidated, admission to inpatient rehabilitation after hospitalization has been found to be significantly higher in patients with pneumonia [67,69,74,76,78-79]. Nosocomial pneumonia in TBI patients is also responsible for a 7-fold increase in odds of lower functional outcomes scores for up to 5 years post-injury [67]. VAP is also found to be associated with survival rates of 46% (for early onset VAP) and 32% (for late-onset VAP) in one-month post-injury while patients without VAP have a survival rate of 69% [69].

Subarachnoid Hemorrhage

Aside from the obvious effects of rebleeding, delayed cerebral ischemia, and hydrocephalus on the brain parenchyma, SAH is associated with other medical complications that play a significant role in patient prognosis after the hemorrhagic event [12]. There is a prominent correlation between pulmonary complications, specifically pneumonia, prolonged LOS, increased morbidity, and increased mortality in patients with SAH.

One of the most important regulatory factors leading to immunodepression and increased infection risk in SAH patients is interleukin 10 (IL-10). Studies show that high blood levels of IL-10 at the time of hospital admission are associated with the occurrence of pneumonia during hospitalization and poor subsequent results at the time of discharge [80]. Risk factors for pneumonia in SAH include older age, lower GCS score, and a history of hypertension [81].

Studies show that aggressive treatment with hypervolemic, hyperdynamic therapies in SAH patients with pulmonary edema has worse consequences and may be due to delayed ischemic neurological deficits. Angioplasty and papaverine infusion seems to have a better effect on the management of pulmonary complications after SAH [1,81].

In patients requiring mechanical ventilation, there is a high incidence of VAP. One single-center retrospective study of patients in a neuro-ICU in France found the following composition of culprit organisms in their early onset VAP cases: MSSA (34.9%), *H. influenzae* (28.1%), *S. pneumoniae* (15.5%), and Enterobacter species (10.7%). Their cases of late-onset VAP were entirely composed of MSSA (53.8%) and Enterobacter species (46.2%). Interestingly, they delineated early and late-onset VAP using a cutoff of pneumonia development preceding or subsequent to 7 days post-intubation. Taking into account the later cut-off, they were able to show a significant correlation between delayed enteral nutrition and the incidence of early onset VAP. Thus they concluded that early enteral nutrition is recommended in SAH patients [82].

Intracerebral Hemorrhage

Pneumonia is the most common nosocomial infection that develops in intracerebral hemorrhage (ICH) patients, and it is associated with a prolonged duration of hospital stay. Furthermore, ICH patients with pneumonia are more prone to undergo the insertion of gastrostomy tubes compared to those who do not have a nosocomial infection. The most important predictor of pneumonia is IVH, but the details of the relationship are not yet entirely known. It is assumed, however, to be due to either more significant immunosuppression inherent in brain injury due to IVH or because the greater risk of hydrocephalus leads to prolonged duration of hospitalization and thus greater risk of development of nosocomial infections [35,48,83-84].

The most common risk factors leading to the development of pneumonia in ICH patients are mechanical ventilation, tube feeding, dysphagia, tracheostomy, age, current smoking, excessive alcohol consumption, COPD, premorbid dependence, ICH severity, infratentorial ICH location, hematoma volume, early hospital admission, in-hospital aspiration, and intubation [85-87].

Status Epilepticus

Status epilepticus can be caused by several infections and can similarly give rise to subsequent infections in the neuro-ICU [88]. About 42% of the patient population have been noticed to develop infections, half of which have been reported as nosocomial [89]. Studies show that VAP is the most common type of infection in patients with status epilepticus in neuro-ICUs with an incidence rate of 48%, and pneumonia is the most common type of infection in the patient population in general [89]. It’s with noticing that patients with prior infections will generally require longer hospitalization and care and as a result, are more susceptible to developing different types of nosocomial infections, with pneumonia being the most common type as mentioned [1]. In addition, up to 80% of the patients have reportedly received thiopentone for seizure control; a medication that can induce an immunosuppressed state and therefore may increase the risk for pneumonia [89]. Interestingly, recent studies show that status epilepticus can happen subsequent to infection with coronavirus disease 2019 (COVID-19) and therefore increase the chance of pneumonia development [90-92].

Spinal Cord Injury

With an incidence rate of 36% to 83%, respiratory complications are responsible for morbidity and mortality in the acute phase of SCI, respectively [93,94]. Patients with a higher level of injury to the spinal cord, for example, at the cervical and thoracic level, are more at risk to develop respiratory complications [95]. Injuries to C1-C4 are significantly associated with mechanical ventilation dependency [96,97]. Three main factors have been observed to be responsible for the development of pneumonia in patients with SCI: retention of secretions because of damaged coughing reflexes, decrease in respiratory muscle strength and fatigue, and autonomic dysfunction [98]. Acute SCI can create immunosuppression due to noradrenergic overactivation and excess glucocorticoid release via the stimulation of the hypothalamus-pituitary-adrenal axis [99]. A study shows that SCI may induce suppression of norepinephrine and an increase in serum cortisol [99]. SCI to the higher thoracic level induces such an effect more significant when compared with the lower levels mainly due to the higher innervation of the adrenal gland [99]. Therefore, the imbalance in glucocorticoids and catecholamine is a key factor in the development of immunomodulation and consequent pneumonia in SCI [99]. Suggested prevention measures in SCI patients have assisted ventilation in patients with impaired ventilatory capacity before it enters an emergency situation, early tracheostomy after short orotracheal intubation, and early weaning [93].

Neuromuscular Diseases

Less tangible neurologic insults are not without their own risk of nosocomial infections. The most common cause for patients with primary neuromuscular diseases to be admitted to ICU settings are involvement of the respiratory muscles [100]. As an example, the characteristic symptoms of myasthenia gravis, the most frequent disorder of the neuromuscular junction, include muscle weakness, fatigue, and dysphagia [101]. This, in turn, can lead to aspiration and subsequent pneumonia formation. It must be noted, however, that this sequence of events is not absolute. The etiology of myasthenia gravis is the pathogenic creation of autoantibodies against certain components of the post-synaptic membrane: the acetylcholine receptor (AChR) or the muscle-specific kinase (MUSK) [101]. One of the most concerning and potentially life-threatening events (and the event that would warrant admission to ICU most frequently) in a patient with myasthenia gravis is the development of a myasthenic crisis (MC). This is defined as neuromuscular respiratory failure requiring mechanical ventilation, and the common preceding trigger is actually respiratory infection [101,102].

Nevertheless, regardless of the actual inciting trigger for the development of MC, failure to extubate and the need to reintubate are fairly common in the setting of neuromuscular diseases in general, which leads to an often prolonged period of mechanical ventilation predisposing to a higher incidence of VAP [100,103]. In turn, the development of VAP can lead to a prolonged duration of intubation in this cohort of patients as was seen in a retrospective study of patients at the University of Miami [104,105]. One multicenter analysis corroborated this statement with the finding that the incidence of pneumonia was lower in cases of MC where noninvasive ventilation (NIV) sufficed compared to cases where intubation and mechanical ventilation were needed. They further postulated that this may be due, in part, to the use of greater amounts of medications, namely sedatives [106]. A retrospective study of patients in an Indian hospital found that, in their MC patients requiring mechanical ventilation, the most common infection noted was pneumonia; comprising 30% of their complications [107]. One study looking into interventions that could improve outcomes for MC patients found that patients who underwent tracheostomy earlier in the course of their MC had a significant decrease in the duration of their need to be mechanically ventilated in an ICU setting, decreased length of ICU stay, and a decrease in their overall hospital LOS [100].

Demyelinating Diseases

Similar to neuromuscular diseases, demyelinating diseases are not without risk of the development of nosocomial infections. In the case of multiple sclerosis (MS), dysphagia can be a common disabling finding and, as stated elsewhere in this review, dysphagia can lead to aspiration and subsequent pneumonia [108]. Although somewhat out of the scope of this paper, the natural course of the disease is not the only predisposing factor to worry about in the case of MS. The disease-modifying treatments themselves increase the possibility of infection. In a study comparing interferon beta-1a to alemtuzumab for the control of MS, it was found that alemtuzumab was associated with an increase in mild to moderate respiratory tract infections [109]. This is not surprising considering the nature of alemtuzumab: it is an anti-CD52 monoclonal antibody that exerts its immunosuppressive effects through a rapid depletion of B and T lymphocytes that then only steadily increase over the subsequent 6-12 months. The phase III trials of the drug, however, demonstrated an uncommon incidence of serious infections, and even the cases of respiratory infection found were mild and non-life-threatening; but, immunosuppression, in general, should cause the clinician to think about opportunistic infections for which the patient may be at risk [110-111]. Interestingly, another phase III trial did not find any serious opportunistic infections, postulating that this may be due to intact innate immunity [112]. Of course, there are always cases that deviate from the expected, as was seen in a case study in which a patient started on alemtuzumab for the treatment of relapsing MS developed ARDS caused by *Pneumocystis jirovecii* and Cytomegalovirus [113]. Of note, organisms identified with causing opportunistic infections are not typically those associated with the hospital- or VAP. Pneumonia has a high incidence in Guillain-Barre patients because of swallowing impairment and aspiration as well as impairment of the respiratory muscles that often leads to early mechanical ventilation. Studies show that the most important risk factor for pneumonia is a long time to intubation since the onset of the disease as an independent factor [114-115].

COVID-19 in neuro-ICUs

The crisis of the COVID-19 pandemic has affected public health in several aspects. In many cases, management and treatment of the patients have been affected because of it and many urgent patients with severe diseases have been neglected because of the situation of hospitals and ICUs and patients’ concerns about hospital admission. This obviously includes patients with neurological diseases [116,117]. Studies show that patients with ischemic stroke, ICH, or SAH that are infected by novel coronavirus have a higher risk of morbidity, mortality, and longer hospital stay. The most common risk factors that play important roles in the prognosis of patients with ICH-COVID and SAH-COVID are obesity, atrial fibrillation, diabetes, and congestive heart failure [118]. In addition, males, younger patients, and Black or Hispanic patients have a higher risk of morbidity and mortality in IS-COVID [119].

Studies show that stroke-COVID demonstrates more systemic complications like acute respiratory failure requiring intubation, acute renal failure, ICH, cerebral venous sinus or deep vein thrombosis, and pulmonary emboli that lead them to higher rates of morbidity and mortality and unfavorable situation upon discharge [119].

Radiographic findings

Imaging plays an important role in the management of pneumonia. In a patient suffering from fever, cough, or sputum production, imaging helps in confirming the diagnosis of pneumonia. However, identification of specific etiological agents is not always possible, since the imaging findings may be non-specific.

Chest radiography (CXR) is the most widely used radiological investigation and in most cases may be the only investigation necessary in treating a patient with pneumonia. The best diagnostic clue is air space opacity or consolidation on CXR associated with purulent secretions, fever >38°C, and leukocytosis or leukopenia. Radiographs usually demonstrate lobar consolidation with air bronchograms with multifocal, patchy, or bilateral appearances [120,121].

Traditionally, routine daily CXR has been performed on ICU patients, particularly those requiring mechanical ventilation. However, the benefit of this practice has recently been questioned. Multiple recent studies have found a low incidence of significant findings in routine radiographs, and no significant difference in mortality, LOS, or ventilator days in patients receiving CXR on a daily basis compared with those receiving chest radiographs for specific clinical indications. The American College of Radiology Thoracic Expert Panel concluded that chest radiographs should be obtained if there is a change in the clinical condition of the patient, or after placement of an endotracheal tube (ETT), central venous pressure, or pulmonary artery catheter, chest tube, or nasogastric tube [122].

**Figure 2:**
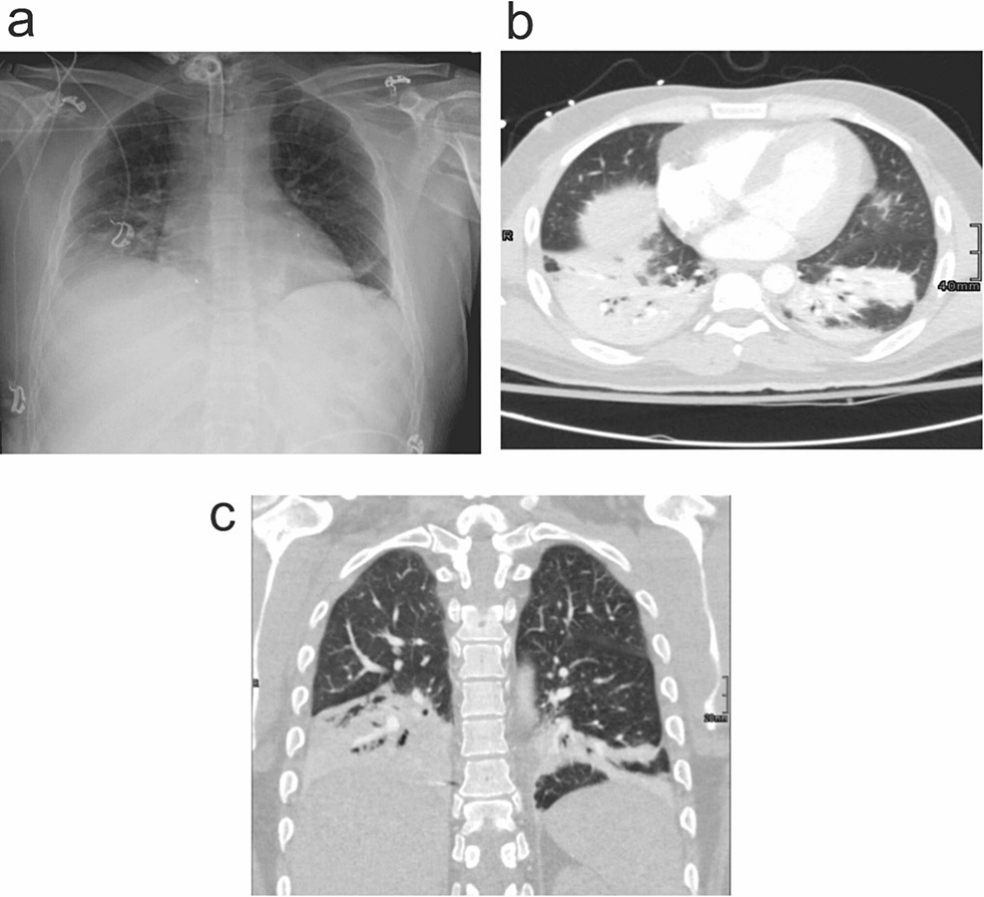
Radiologic findings in patients with nervous system injuries and developing pneumonia a. The radiograph demonstrates right basilar pleuroparenchymal and left basilar linear airspace opacities. No large pleural effusion or pneumothorax is identified. b. Axial view of the CT chest with contrast which was performed the next day for evaluation of a possible pulmonary embolism shows bibasilar consolidative opacities with air bronchograms. No segmental or sub-segmental pulmonary embolism was identified. c. Coronal view of the same patient in b.

It is necessary to use cross-sectional imaging modalities like computed tomography (CT) due to atypical or non-specific chest radiograph findings in pneumonia cases. CT may be useful in selected cases, and findings usually are more extensive than on radiography and include segmental, sub-segmental, or scattered patchy peribronchovascular consolidation (i.e., airspace disease). CT usually narrows down the differential diagnosis, for etiological agents. It also helps in the evaluation of the causes of non-resolving pneumonia, pulmonary, and non-pulmonary complications of pneumonia. Mentioned radiological findings can be seen in Figures 2a-2c.

Predictors of pneumonia

Considering the worsened outcomes for patients and the extensive costs imposed on the healthcare system through increased lengths of stay and longer duration of ICU care, it becomes important to be able to predict which patients are more likely to develop nosocomial pneumonia. A retrospective study of several tertiary care facilities in Minnesota identified the following as risk factors for pneumonia development in the subset of patients with intracranial hemorrhage: age, race, time of symptom onset in relation to arrival at the hospital, presence of IVH, aspiration, intubation, nasogastric tube placement, evacuation of hematoma, and tracheostomy placement [87]. In the case of TBI, one meta-analysis found a significant relationship between VAP and patient smoking status, blood transfusion on admission, tracheostomy, infusion of barbiturate, and increased head abbreviated injury scale (AIS) [69]. After deciding that a patient is at particular risk, the focus then shifts to what signs and symptoms can be used to detect the development of pneumonia at the earliest point in the course of the disease.

To this end, much has been made of the use of PCT as a marker of bacterial infection. However, numerous studies have now cast doubt on its use as a sole biomarker indicative of bacterial infection. The current CAP guidelines do not recommend the use of PCT to determine the initial need for antibiotics in the empiric treatment of pneumonia [123]. In line with these guidelines, a study conducted on ICU patients attempting to use PCT to differentiate between aspiration pneumonia and aspiration pneumonitis concluded that PCT had poor diagnostic performance in ruling out pneumonia [53]. Because of these studies, the consensus remains that PCT may be used as a tool to aid in the de-escalation of antimicrobial therapy, but should not be used as the sole factor in determining the need for starting antibiotics.

Prevention

Having mentioned most of the risk factors for pneumonia in the NICU, addressing dysphagia is one of the most important interventions [124]. Improved screening for dysphagia and nurse education has been shown to decrease the risk of pneumonia as was demonstrated in a single-center study which showed a decrease in pneumonia prevalence from 6.5% to 2.8% after the screening and education changes were implemented. Early administration of prophylactic antibiotics has not been shown to be effective in decreasing mortality or functional outcome in these patients and is not indicated [125]. Likewise, oropharyngeal decontamination with povidone-iodine has not been effective in the prevention of VAP in patients with critical brain injuries or cerebral hemorrhages [72]. Contrarily, employing such methods may increase the occurrence of ARDS and brings into question the safety of such measures [72].

The timing of tracheostomy placement in such patients who require continuous ventilation does not seem to affect the mortality rate; however, it has been indicated that early tracheostomy placement may decrease the duration of ventilation [126,127]. Early extubation is strongly associated with a lower risk of pneumonia development [127,128].

The utilization of temperature management has also been a popular research avenue for the prevention of pneumonia. Maintenance of normothermia has shown promise as a measure to attenuate secondary brain injuries after the initial insult; however, prolonged targeted temperature management beyond 72 hours was shown to significantly increase the incidence of VAP due to hypothermia-induced immunosuppression [68]. Although corroborated in a subsequent meta-analysis, the higher risk of VAP in TBI patients who received targeted temperature management was not observed in a case-control study utilizing normothermia in SAH patients [128-129].

Another prevention strategy is addressing brain injury-induced immunosuppression. As mentioned, this immunosuppression is mainly due to sympathetic nervous system activation. The use of β-adrenergic receptor blockers was demonstrated to attenuate the weakening of the immune system and reduce the risk of subsequent infections such as pneumonia [37]. The administration of β-blockers has also been effective in decreasing the risk of early death in patients with stroke and increasing survival in patients with TBI [130-133]. Nevertheless, further assessment of β-blocker administration needs to be carried out in order for it to be confirmed as a routine choice for the prevention of pneumonia in NICUs [1].

Last but not least, collaborative and multidisciplinary approaches can lead to a significant decrease in nosocomial infections [24]. Empowerment of respiratory therapists and nurses, an oral care regimen, patient positioning protocols, subglottal suction for intubated patients, and aggressive antibiotic stewardship are all major effective approaches that can make a huge impact on the prevention of pneumonia [134].

Treatment

Despite the separation and resultant classification of pneumonia as being either hospital-associated or ventilator-associated and the type of brain injury preceding its development, the treatment progresses along with the same algorithm. In all cases of HAP or VAP, the empiric treatment course should be with an antibiotic regimen that will cover both *S. aureus* and *P. aeruginosa*. Whether to use an agent with activity against MRSA depends on 1) if the patient has risk factors for antimicrobial resistance, 2) if the prevalence of MRSA in that particular ICU exceeds 10-20% (determined by institution-specific antibiogram), or 3) if the prevalence of MRSA in that setting is unknown [73,135]. In regards to what constitutes the increased risk for antimicrobial resistance in a patient, it is the administration of previous IV antibiotics within the preceding 90 days. Similar to the criteria governing the choice of antibiotics with MRSA coverage, the use of a double coverage strategy of two antibiotics from different classes for empiric coverage of *P. aeruginosa* is dependent on several criteria being met: 1) If, according to the institution-specific antibiogram, greater than 10% of gram-negative isolates exhibit resistance to the agent being considering for monotherapy, 2) If the patient is being treated in a facility where no antibiogram data is available and thus antimicrobial susceptibility rates are unknown, 3) If the patient has a high risk for mortality (requirement of ventilatory support, presence of septic shock), or 4) The patient has structural lung disease at baselines such as severe emphysema, bronchiectasis, or cystic fibrosis [73]. Lowering the diagnostic threshold of VAP by strictly adhering to pulmonary signs and symptoms, such as a change in sputum character, oxygen desaturation, new or worsening tachypnea, or new infiltrates on chest X-ray is also crucial as it can prevent prolonged prescription of antibiotics and increased health costs in addition to the drug reactions and development of resistance to antibiotics [136].

In summary, unless the preceding criteria are met to warrant administration of an anti-MRSA agent or the use of double anti-pseudomonal coverage, an identified case of HAP or VAP should be treated with agents that preliminarily at least cover *P. aeruginosa* and MSSA. Examples of such regimens that would be used in patients without any risk factors for multi-drug resistant organisms (MDR) include monotherapy with piperacillin-tazobactam 4.5 g IV every 6 hours, cefepime 2 g IV every 8 hours, or levofloxacin 750 mg IV daily. If, however, the criteria are met to start empiric coverage for MRSA (vancomycin or linezolid), then cultures and/or nasal PCR should be done to allow for de-escalation of antibiotics as warranted. Mirroring this strategy, if coverage is started for *P. aeruginosa*, then respiratory culture should be done to allow for de-escalation of therapy. Example regimen composition for double coverage of *P. aeruginosa* includes a beta-lactam or carbapenem antibiotic coupled with respiratory fluoroquinolone or aminoglycoside. Of note, if *P. aeruginosa* is identified as the culprit pathogen, then the patient’s clinical status should be used to guide whether to continue the double coverage or to deescalate to monotherapy [137]. In general, in the name of antimicrobial stewardship, it is suggested that antibiotics be de-escalated to the least broad antibiotics capable of adequately treating the infection. The treatment duration should generally extend no longer than 7 days once appropriate coverage is obtained [138,139]. A summary of common pathogens and preferred treatments for different types of injury can be seen in Table 3.

**Table 3:** Pneumonia in common neurocritical conditions NIHSS: NIH Stroke Scale; MCA: middle cerebral artery; GCS: Glasgow Coma Scale; CNS: central nervous system; CSF: cerebrospinal fluid; COPD: chronic obstructive pulmonary disease [16,55,65-66,69,72,81-82]

CNS Condition	Risk Factors for Pneumonia	Common Pathogens	Antibiotics
Stroke	Dysphagia, higher NIHSS, non-lacunar basal-ganglia infarction, older age, large MCA infarction, multiple hemispheric or vertebrobasilar infarction mechanical ventilation on admission	Gram-negative bacilli	Ceftriaxone
			Levofloxacin
			Moxifloxacin
			Ciprofloxacin
			Ampicillin/Sulbactam
			Ertapenem
			Piperacillin-Tazobactam
		Staphylococcus aureus	Ceftriaxone
			Levofloxacin
			Moxifloxacin
			Ciprofloxacin
			Ampicillin/Sulbactam
			Ertapenem
			Piperacillin-Tazobactam
		Anaerobic bacteria	Ampicillin/Sulbactam
			Ertapenem
			Piperacillin-Tazobactam
Subarachnoid hemorrhage	Prolonged length of stay, older age, lower GCS score, history of hypertension	Methicillin-susceptible *Staphylococcus aureus*	Ceftriaxone
			Levofloxacin
			Moxifloxacin
			Ciprofloxacin
			Ampicillin/Sulbactam
			Ertapenem
			Piperacillin-Tazobactam
		Haemophilus influenza	Ceftriaxone
			Levofloxacin
			Moxifloxacin
			Ciprofloxacin
			Ampicillin/Sulbactam
Traumatic brain injury	Surgical intervention, prolonged hospitalization, damage to the CNS, CSF leak, nasal carriage of *S. aureus*, use of barbiturate, need for intubation and mechanical ventilation, male gender, younger age, early intubation, lower GCS score, longer mechanical ventilation, higher NIHSS injuries to thorax	Methicillin-susceptible *Staphylococcus aureus*	Ceftriaxone
			Levofloxacin
			Moxifloxacin
			Ciprofloxacin
			Ampicillin/Sulbactam
			Ertapenem
			Piperacillin-Tazobactam
		Haemophilus influenza	Ceftriaxone
			Levofloxacin
			Moxifloxacin
			Ciprofloxacin
			Ampicillin/Sulbactam
		Streptococcus pneumoniae	Ceftriaxone
			Levofloxacin
			Moxifloxacin
			Ciprofloxacin
			Ampicillin/Sulbactam
			Ertapenem
			Piperacillin-Tazobactam
		Acinetobacter species	Ceftazidime
			Ampicillin/Sulbactam
			Meropenem
Intracerebral hemorrhage	Mechanical ventilation, tube feeding, dysphagia, tracheostomy, older age, current smoking, excessive alcohol consumption, COPD, ICH severity, infratentorial ICH location, hematoma volume, early hospital admission, intubation	Methicillin-susceptible *Staphylococcus aureus*	Ceftriaxone
			Levofloxacin
			Moxifloxacin
			Ciprofloxacin
			Ampicillin/Sulbactam
			Ertapenem
			Piperacillin-Tazobactam
		Haemophilus influenza	Ceftriaxone
			Levofloxacin
			Moxifloxacin
			Ciprofloxacin
			Ampicillin/Sulbactam

## Conclusions

Patients with nervous system injuries are susceptible to developing infections with pneumonia being the most common type. The activation of the sympathetic nervous system in response to brain injury gives rise to a state called brain injury-induced immunosuppression which, in turn, makes the patients more prone to infection. Risk factors such as prolonged LOS in neuro-ICU, early and prolonged mechanical ventilation, and absence of swallowing reflex among other risk factors can facilitate the process of pneumonia development. Injuries such as ischemic stroke, SAH, ICH, and TBI are the most common types responsible for developing pneumonia in neurocritical patients. A multidisciplinary approach including efficient education of staff, management of dysphagia, strict infection control protocols, temperature management, and addressing the brain injury-induced immunosuppression can lead to a significant decrease in respiratory complications and nosocomial infections in general. For future perspective, a thorough study of brain injury-induced immunosuppression and assessment of β-blocker administration may introduce new therapeutic approaches and firm steps toward infection control in CNS injuries.
